# Research on the rapid combustion process of butane under microwave discharge

**DOI:** 10.1038/s41598-021-04021-0

**Published:** 2022-01-07

**Authors:** Qiang Tang, Zhibin Hu, Zechao Tao, Dan Ye, Jau Tang

**Affiliations:** Institute of Technological Sciences, Wuhan University, Wuhan, 430072 Hubei China

**Keywords:** Natural gas, Plasma-based accelerators, Imaging techniques

## Abstract

To study the combustion process of fuel in the microwave plasma torch, we designed a butane microwave plasma device exploiting a tungsten rod as an electrode. Through analysis of the image record by high-speed camera, we found that the discharge of butane microwave plasma torch is a cyclic process at atmospheric pressure at a frequency  of around 100 Hz. During the discharge, the active particles continuously diffuse from the electrode to the outside like the bloom of the flower. Then, the variation of plasma torch of jet height and temperature with microwave power is obtained. In addition, we studied the effects of different butane flow rates on the plasma torch. The results illustrate that excessive butane will lead to carbon deposition on the electrode. All in all, this work provides a new understanding of the combustion of the microwave plasma torch, which is conducive to the further development of microwave plasma in the fields of waste gas treatment, fuel combustion, and plasma engine.

## Introduction

Using electromagnetic energy and non-equilibrium plasma-assisted combustion to improve thermal energy conversion has great potential and can be used as a foundation for improving energy usage^1,2^. The application of electromagnetic energy to combustion reactions has many benefits. For instance, it can achieve faster and stronger chemical energy conversion, improve combustion stability, improve fuel efficiency, provide a stable fuel oxidation range, and generate higher pressure and temperature which cannot be easily produced by regular methods^2–6^. Using this way can also reduce pollution by changing the oxidation by-products and providing a faster and more reliable ignition method either^7,8^. When the electromagnetic energy acts on the flame, it can decompose the fuel from the larger hydrocarbon to the smaller hydrocarbon, and collide with electrons to excite radicals^9,10^.

The effects of different discharge systems on combustion have been investigated by various research groups, including microwave, direct current, radio frequency, etc.^11–14^. The advantages of a microwave plasma torch in combustion are its role as a source of a radical pool and high temperature^15–17^. Since the 1970s, there are many experimental works related to fuel combustion using plasma torch^18–21^. For instance, Yong et al.^20^ developed a microwave plasma burner by injecting hydrocarbon fuel in liquid or gaseous state into the microwave plasma torch generated by air, which found the increase of fuel can greatly increase the temperature of plasma torch. And Takita et al.^22^ showed the number of radicals and electron temperature can increase the combustion rate of fuel. To further explore the combustion principle of microwave plasma torch, it is essential to understand the process of plasma torch exciting fuel^23–28^.

Here, a microwave device with an electrode is designed, and the high-speed combustion process of fuel in microwave discharge is studied by taking butane as an example. The rapid periodic discharge law and combustion state of the butane plasma torch with different conditions are found in this device. When butane is excited, the active particles continuously diffuse from the electrode to the outside like the bloom of the flower. These phenomena show a new comprehension of microwave combustion support.

## Experimental setup

**Figure 1:**
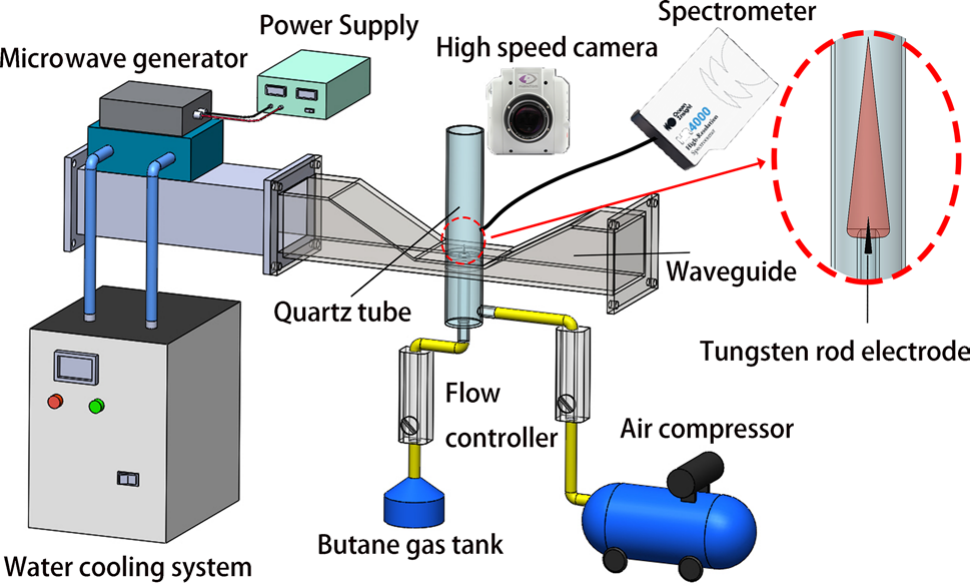
Schematic of the experimental setup.

Figure 1 shows a schematic view of the butane microwave plasma torch system. It consists of the 2.45 GHz microwave generator, waveguide components, gas supply, a tungsten rod electrode and plasma torch detection device. The microwave power supply provides a maximum power of 1000 W and is connected to magnetron (LG, M246, South Korea) to generate microwaves. The water cooling system is a circulating water tank connected to microwave generator. The waveguide cavity is composed of rectangular waveguide (WR340) and dual-port tapered waveguide^29^. A quartz tube is inserted vertically at the opening of the upper and lower surfaces of the dual-port tapered waveguide to guide the gas flow and produce plasma torch. The quartz tube has an inner diameter of 24 mm, an outer diameter of 28 mm and a length of 600 mm. The gas supply system is mainly composed of an air compressor, butane gas tank and two flow controllers (KONGXINYIBIAO, 700C-D10W, China). The butane and air pass through the flow controllers and enter the inner and outer quartz tubes from the two inlets. The plasma torch detection device includes a high-speed camera (Phantom, V1612, USA) matching with a microscopic zoom lens (Navitar 12-X), an optical emission spectroscopy (Ocean Optics, HR4000CG-UV-NIR spectrometer) and an infrared thermometer (WAHOME, IS-CF2000AD, China).

**Figure 2:**
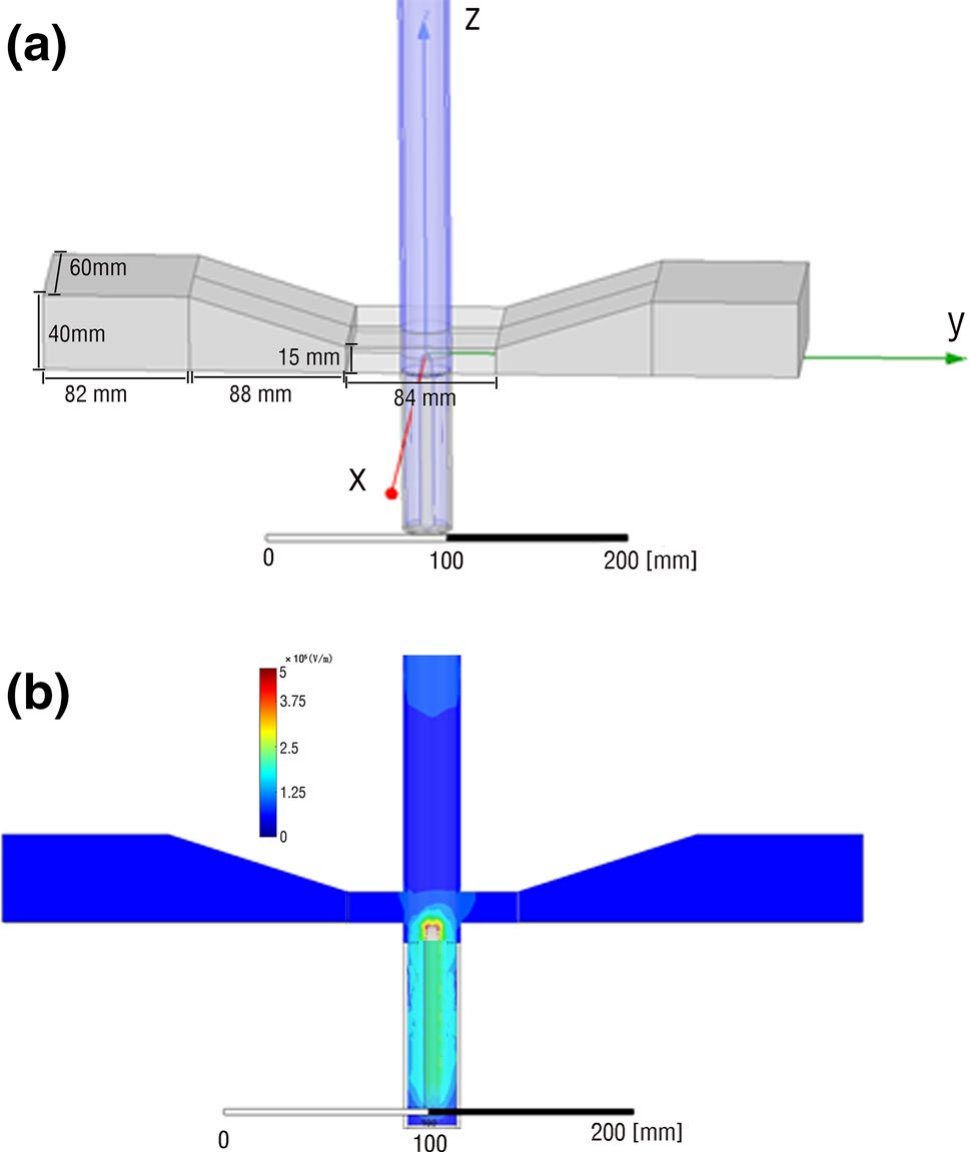
**(a)** 3D model of waveguide in HFSS. **(b)** Microwave discharge cavity electric field distribution image of HFSS.

To verify the rationality of the device structure and obtain the parameters more beneficial to the discharge, the electric field in the discharge chamber is simulated by HFSS. Firstly, the waveguide is modeled, as shown in Fig. 2a. The waveguide is symmetrical on both sides. The length of the rectangular waveguide section is 82 mm, the length of the transition section is 88 mm, the length of the compression section is 84 mm, and the height of the compression end is 15 mm. The diameter of the tungsten rod electrode is 2 mm. The excitation source is the left side of the graded waveguide as Wave Port, and the input power is set to 100 W. Finite Conductivity is applied to the solid wall boundary condition. At the same time, because the waveguide is made of metal material, except for the wave port, the other waveguide surfaces are set as finite conductor boundary conditions, and the solution frequency is set to 2.45 GHz. After setting, the simulation calculation is carried out, as shown in Fig. 2b. The results show that the electric field intensity peak appears in the area where the tungsten rod electrode is close to the quartz tube, and the region with a strong electric field is mainly concentrated at the top of the tungsten rod. The maximum value of the electric field is 5.04 × 10^5^ V/m. It can be seen that the tungsten rod electrode effectively couples the microwave electromagnetic energy to the discharge chamber, which is also in line with the original design intention of the experimental structure.

**Figure 3:**
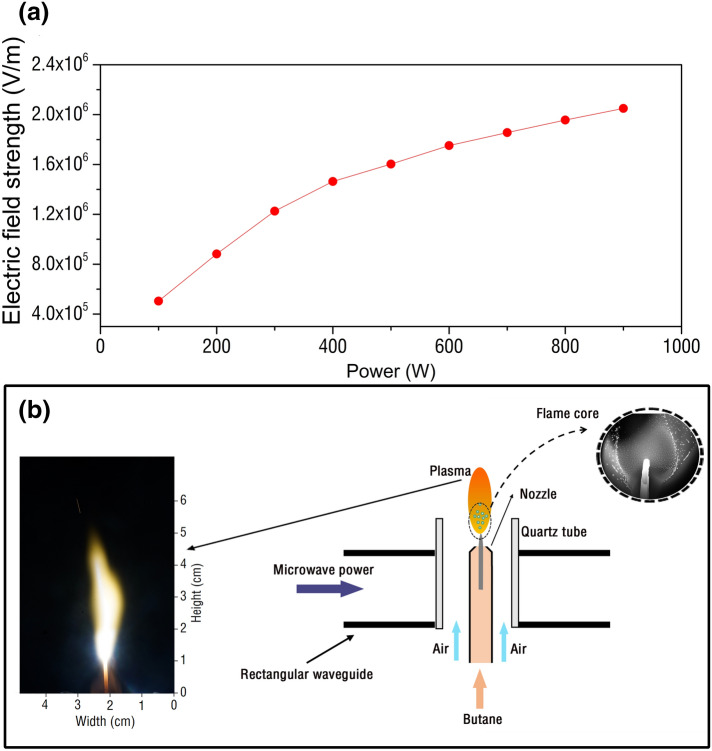
**(a)** Maximum electric field intensity generated by microwave plasma device at different power. **(b)** Butane fire excited by microwave discharge, the left is the actual image of the torch flame and the upper right is the image of the flame core.

However, the electric field strength under the power of 100 W is not enough to break down the butane flame and form the plasma torch. The breakdown field for flame in the air can be calculated as^30^:

1 $$\begin{array}{*{20}c} {E = 2.57 \times 10^{6} \frac{{T_{r} }}{{T_{g} }}\left( {V/m} \right)} \\ \end{array}$$

where ${T}_{r}=300 \mathrm{K}$ is the room temperature and ${T}_{g}$ is the core temperature in butane flame, around 800 K. The electrical breakdown in room-temperature air occurs at the electric field of 2.57 × 10^6^ V/m. The breakdown electric field in flames is inversely proportional to the flame temperature ${T}_{g}$. Therefore, we can produce plasma very easily by focusing relatively low power microwave into the high temperature flame. The breakdown field for butane flame is 9.63 × 10^5^ V/m. According to the variation of the maximum electric field of microwave plasma device under different power (Fig. 3a), the minimum power of the device can be obtained as 300 W. The butane plasma torch excited by microwave discharge can be seen in Fig. 3b. The large quartz tube is placed in the center of the waveguide, and a small quartz tube flame nozzle is also set in the large quartz tube. Butane gas is introduced into the quartz tube flame nozzle, and a large flow of air is introduced between the large quartz tube and the small quartz tube flame nozzle. Under the action of microwave, butane molecules are continuously excited at the tip of tungsten rod to produce active particles. A bright flame appeared above the tungsten rod.

## Results and discussion

**Figure 4:**
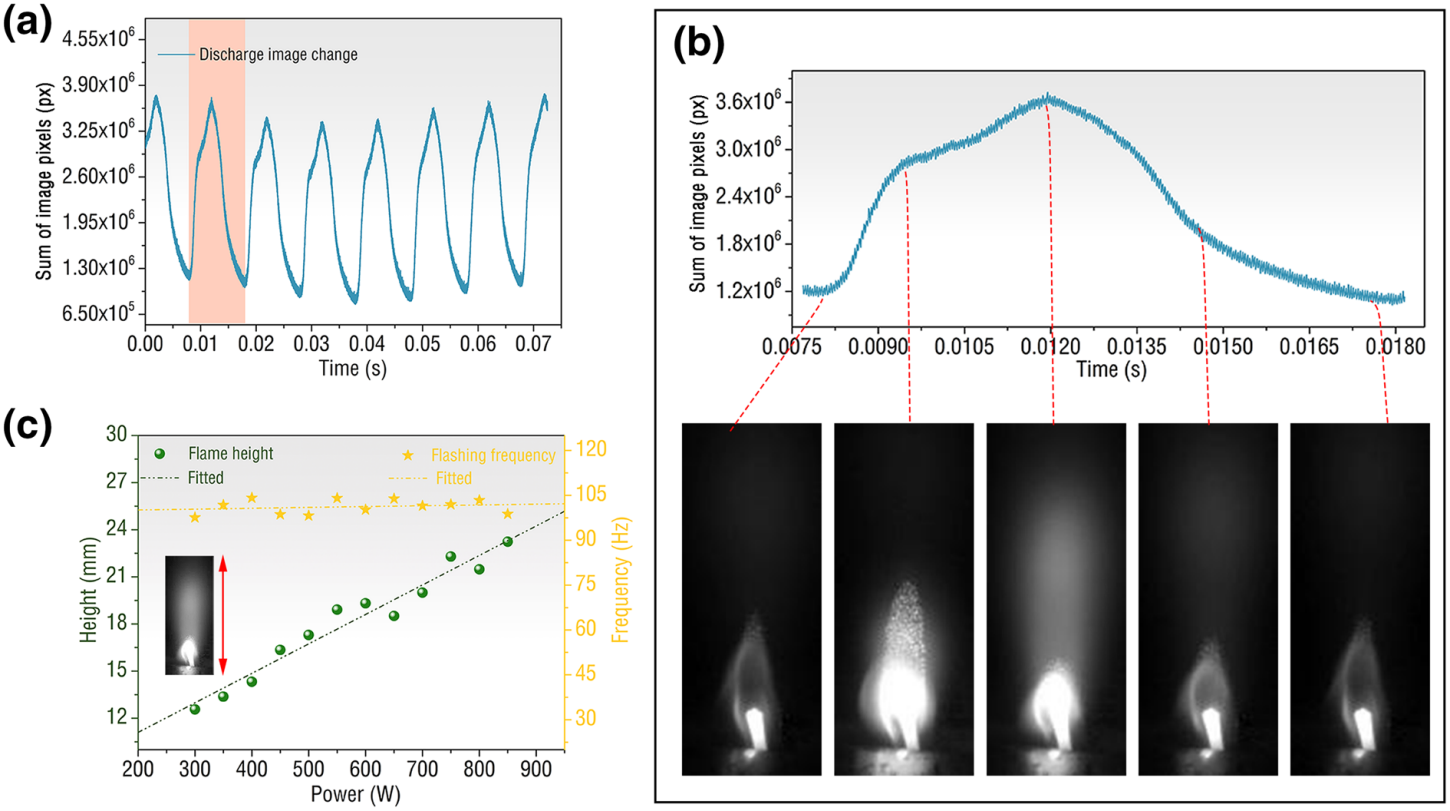
Characteristics of the butane microwave plasma torch with butane and air flow rates in 30 SCCM and 1.2 m^3^/h, respectively. **(a)** The change of sum of pixels of the microwave discharge image over time (multimedia view: supplementary video 1). **(b)** The change of sum of pixels of the microwave discharge image in one cycle. **(c)** The maximum jet height and flash frequency of butane microwave plasma torch vary with power.

At the beginning of the experiment, the flow rate of butane was set to 30 SCCM and the air flow rate was set to 1.2 m^3^/h, and the power of the microwave was adjusted to 800 W. When the discharge starts, a high-speed camera is used to record the discharge image of the flame with 130,000 frames. By summing the pixels of discharge images of each frame, the light and dark changes of the flame can be obtained. The sum of image pixels change over time is shown in Fig. 4a. From the figure, we can see that the discharge of butane under the action of microwave plasma exhibits obvious periodicity, with a period of about 0.1 s. The specific discharge image is shown in Fig. 4b. It shows the discharge combustion of butane in one discharge cycle. In the first half of the cycle, the combustion of butane microwave plasma torch is to inject high-energy particles upward from the core of the discharge. After the spray reaches the highest height, it enters the second half of the discharge cycle. In the second half of the cycle, the high-energy particles injected by butane gradually disappear, and finally return to the state when the injection started, waiting for the next injection. When the constant power increases from 0, the change of plasma torch can be found, as shown in Fig. 4c. The minimum power of the excited butane plasma torch is 300 W. With the increase of power, the maximum jet height of the torch increases linearly. By contrast, data not shown the torch injection frequency hardly changes with the power and tends to be stable. The torch jet frequency fluctuates between 95 and 105 Hz and is generally centered at approximately 102 Hz.

**Figure 5:**
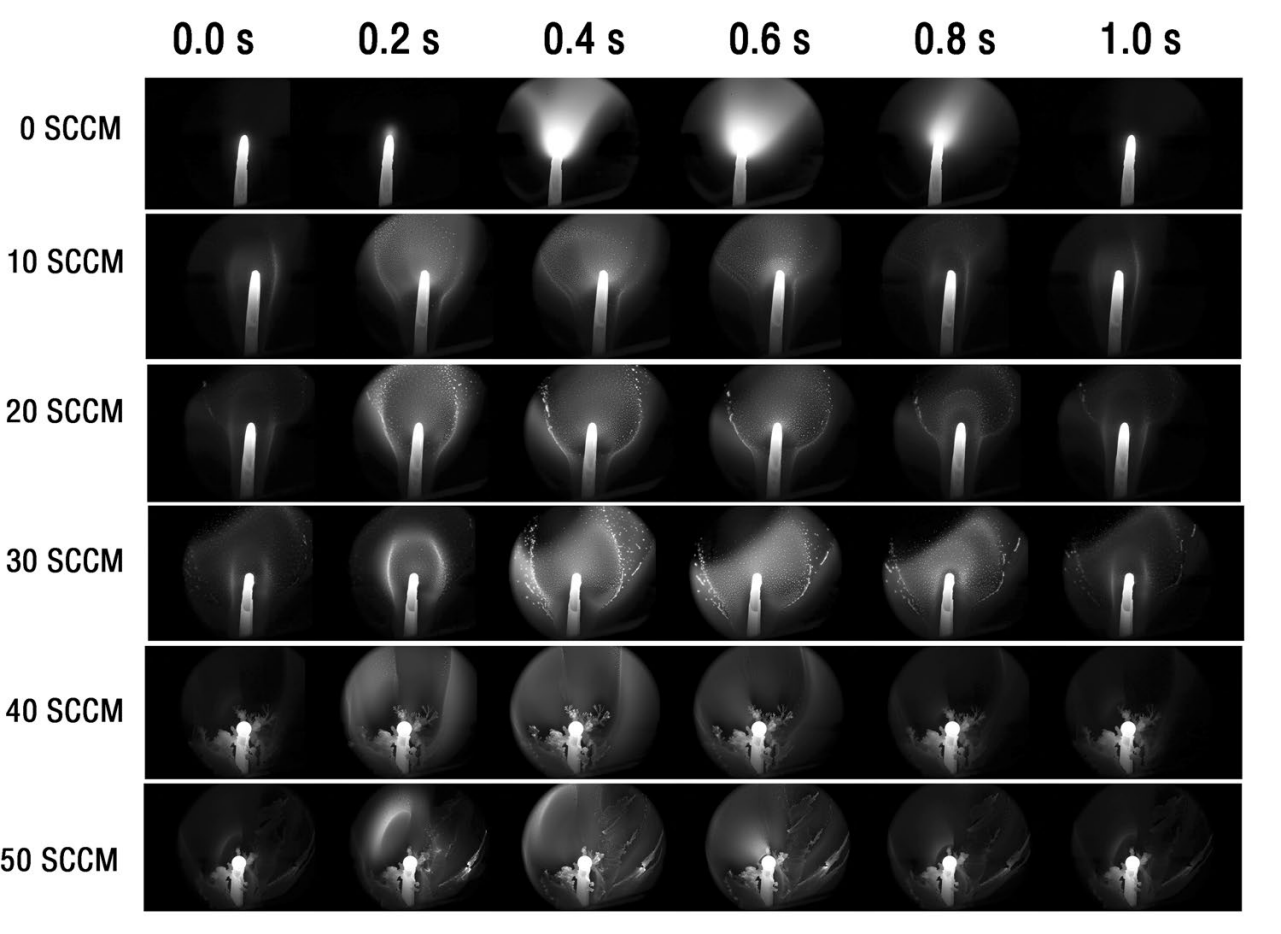
Butane microwave plasma discharge on tungsten rod captured by a high-speed camera with different butane flow rates. (multimedia view: supplementary video 2 and supplementary video 3).

From the previous paragraph, it is obvious that when butane is burned in a microwave plasma torch, high-energy particles will be continuously injected from the discharge core, and the frequency does not change with the power. Thus, we fixed the power to 800 W and changed the butane combustion flow to find out the changes at the core of the butane combustion discharge, as shown in Fig. 5. When the butane flow rate is zero, the air microwave plasma is formed only by the tungsten electrode with the action of the microwave. At this time, the microwave plasma does not produce active particles ejected into the air. At 10 SCCM, because butane gas began to participate in the reaction, the microwave plasma discharge was more intense, and many active particles began to be produced at the same time. Continue to increase the flow of butane gas to 20 SCCM. At this time, a bright, approximately circular, large active particle band has been generated around the tungsten rod, like a flower. When the gas flow rate reached 30 SCCM, the large active particles surged and reached a peak, and some active particles are connected into threads to form smaller carbon deposits. This result shows that the amount of butane excited by microwave is limited at a certain microwave power, and the excess butane will be converted to solid carbon. When the inflow of butane reaches 40 SCCM, the carbon deposition is more obvious, and a small amount of flocculent carbon deposition is produced around the tungsten electrode. In contrast, almost all linear carbon deposits are transformed into flocculent carbon deposits at 50 SCCM, which shows that the combustion is very inadequate.

**Figure 6:**
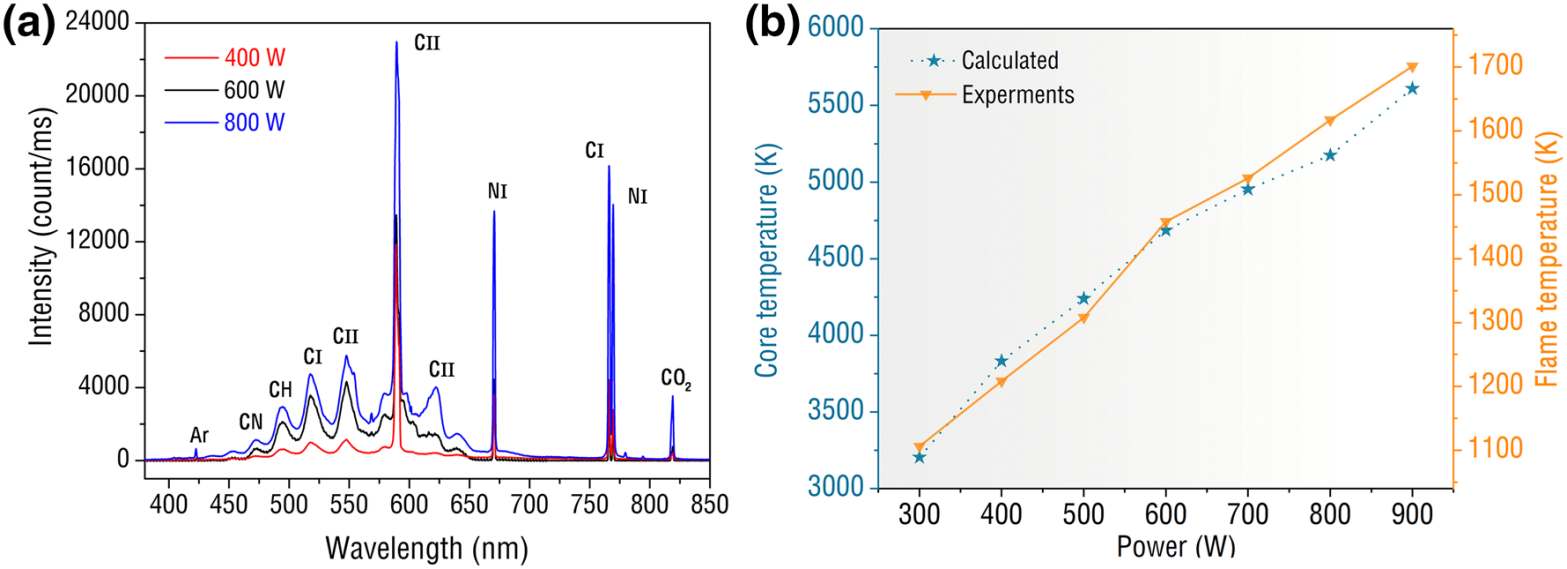
**(a)** Butane microwave plasma torch excitation spectrum image. **(b)** Butane microwave plasma outer flame and inner core temperature change with power.

In order to understand the combustion of butane microwave plasma torch in more detail, the emission spectra of butane microwave plasma torch are collected and analyzed. Plasma spectrum detection equipment is set on the waveguide, the spectrum probe is 20 cm away from the torch, and the collected torch spectrum image is shown in Fig. 6a. The abscissa represents the spectral wavelength, and the ordinate represents the relative intensity. This is an experiment conducted under microwave power of 400 W, 600 W and 800 W. Many CI and CII elements are excited between 500 and 600 nm, and only a small amount of CH bonds is excited at a wavelength of about 500 nm.

In addition, there are also electronic excitations of other species in flame, including the CO_2_ and the OH (300–400 nm)

2 $$\begin{array}{c}CO+O+M\to C{O}_{2}^{*}+M\end{array}$$

3 $$\begin{array}{c}O+H\to O{H}^{*}\end{array}$$

Which has been proved by many researchers^30,31^. These elements are excited because oxygen molecules are converted into atoms by the electron attachment during electrical breakdown. Oxygen atoms are the most reactive radicals in flames, and the abundant oxygen atom generation may be most beneficial to the oxidation, which makes butane burn completely. Specific reaction process can be expressed by the following formula^32^:

4 $$\begin{array}{c}{O}_{2}+{e}^{-}\to O+O+{e}^{-}\end{array}$$

5 $$\begin{array}{c}{C}_{4}{H}_{10}+10O\to 10OH+4C\end{array}$$

6 $$\begin{array}{c}C+2O\to C{O}_{2}\end{array}$$

The combustion process of butane is also accompanied by extremely high temperature, as shown in Fig. 6b. The abscissa is the microwave power and the ordinate is the temperature of the core of the butane microwave plasma and the outer flame, respectively. The outer flame of the butane microwave plasma torch was measured by an infrared thermometer to obtain experimental data. The minimum/maximum temperature of the outer flame is 1106 K and1701 K at the microwave power of 300 W and 900 W. The temperature of the outer flame of the torch increases with the increase of the microwave power, and the relationship is approximately linear. As for the core temperature of butane microwave plasma, since traditional methods cannot be used for measurement, the spectral temperature measurement method based on the slope method is used for calculation^33^. The core temperature is 3205 K when 300 W microwave power is obtained, and the core temperature surges to 5611 K when it reaches 900 W. The core temperature of the torch is also positively correlated with the microwave power. In summary, whether it is the outer flame temperature or the core temperature, they all increase with the increase of the microwave power, and both indicate that the butane microwave plasma burns violently and fully.

## Conclusions

In this research, a microwave plasma device with electrodes is proposed that is used to test the combustion of butane under different conditions. The characterizations of the butane microwave plasma torch have been conducted using digital imaging and optical emission spectrum. The butane microwave plasma torch has been investigated at different fuel equivalence ratios and various microwave power. The digital imaging results show that the plasma regularly discharges at a frequency of about 100 Hz and continuously excites the active particle to jet upward. And there is a linear relationship between active particle jet height and power. Further analysis of the electrode discharge shows that the active particles are generated near the tungsten rod and then diffuse outward, just like a flower. When butane at 40 SCCM and 50 SCCM flow rates is burned at 800 W microwave power, the fuel could not be fully excited by microwave, and flocculent carbon deposition is produced around the tungsten electrode. The optical emission spectrum results show that the CI, CII, and CH emission intensity peaks can be observed in the butane plasma torch. And the maximum temperature at the core flame and outer flame can reach 5611 K and 1701 K at 900 W, respectively. This work would be helpful in further explorations of microwave plasma combustion and develop an optimal scheme for waste gas treatment, fuel combustion, and plasma engine.

## Supplementary Information

Supplementary Video 1.

Supplementary Video 2.

Supplementary Video 3.

Supplementary Legends.
