# Supplementary material for: Research on the rapid combustion process of butane under microwave discharge
Source: Sci Rep. 2022 Jan 7;12:10. doi: 10.1038/s41598-021-04021-0 (PMC8741766; doi:10.1038/s41598-021-04021-0)
Supplement: Supplementary file 4 — Supplementary Legends. [file 41598_2021_4021_MOESM4_ESM.docx]

Supplementary Materials for

**Research on the rapid combustion process of butane under microwave discharge**

Qiang Tang, Zhibin Hu, Zechao Tao, Dan Ye, Jau Tang

Institute of Technological Sciences, Wuhan University, Wuhan, Hubei 430072, China

**This file includes:**

Legends for movies S1 to S3.

**movie S1. Periodic discharge of butane microwave plasma torch at 800W:** periodic discharge image of torch with high-speed camera under the flow rate of butane was 30SCCM.

**movie S2.** **Excitation of active particles near the electrode at 800W under the flow rate of butane was 30SCCM:** the active particles excited by microwave are produced on the electrode, and then radiate outside, like flowers open.

**movie S2.** **Carbon deposition near the electrode at 800W under the flow rate of butane was 40SCCM:** the active particles near the electrode gradually form carbon chains and grow on the electrode.
